# Exploration of biomarkers of Alzheimer’s disease based on orthogonal multi-task canonical correlation analysis

**DOI:** 10.1186/s12880-025-01782-2

**Published:** 2025-07-17

**Authors:** Tao Yang, GuangYu Wan, Xiong Zhou

**Affiliations:** Department of Radiology, Xianning Central Hospital, No. 228 Jingui Road, Xianning, China

**Keywords:** Alzheimer's disease, Image genetics, Canonical correlation analysis, Orthogonal constraint, Biomarkers

## Abstract

As a neurodegenerative disease, Alzheimer’s disease (AD) has many symptoms, such as memory impairment, cognitive decline, and personality change. Image genetics is the correlation analysis between imageology and genetics, and image genetics research can effectively detect the biomarkers of AD. This paper proposed an orthogonal multi-task sparse canonical correlation analysis (MTOSCCA) algorithm. Based on the multi-task canonical correlation analysis, this algorithm added orthogonal constraints to the weight vectors U and V, which can effectively prevent the influence of redundant features on the results. In this paper, the MTOSCCA algorithm was applied to structural magnetic resonance imaging, single nucleotide polymorphism, and gene expression data integration. The results showed that the proposed algorithm has better correlation performance, and the obtained markers have diagnostic significance for AD and mild cognitive impairment (MCI).

**Supplementary Information:**

The online version contains supplementary material available at 10.1186/s12880-025-01782-2.

## Introduction

As a severe neurodegenerative disease, the incidence of Alzheimer’s disease (AD) is increasing yearly [1]. AD patients will suffer from memory impairment and cognitive decline. In severe cases, personality changes will occur [2]. At present, there is no effective treatment for AD. Mild cognitive impairment (MCI) is a state of transition to AD, in which patients will have symptoms such as cognitive impairment. A large number of studies have shown that the occurrence of AD is closely related to genetic variation, and several AD risk genes have been confirmed [3]. It is essential to explore the pathogenesis of AD at the molecular level.

Image genetics can take all kinds of brain images as phenotypes and use various correlation analysis methods to dig out the molecular information hidden in brain images and then build a bridge between macro and micro so as to identify the biomarkers of AD more accurately [4]. Sparse canonical correlation analysis is a powerful correlation analysis algorithm that can maximize the correlation between two or more modal data and get the weight of each feature of each data. Fang et al. developed a joint SCCA algorithm, which uses diagnostic tag information to identify SNP- functional magnetic resonance imaging association patterns with consistent and specific diagnostic categories [5]. Du et al. proposed a multitask SCCA(MTSCCA) algorithm and studied the relationship between structural magnetic resonance imaging, positron emission tomography, and single nucleotide polymorphism (SNP). In addition, they integrated MTSCCA and logistic regression (LR) into a model and embedded the diagnostic information of AD into the model through LR. The objective function maximized the canonical correlation coefficient and the classification accuracy of LR and transformed the original unsupervised SCCA into a supervised SCCA [6].

In this paper, the MTSCCA algorithm was further innovated, and orthogonal constraints on U and V were added to help the algorithm select features. We applied the proposed algorithm to the association analysis of the region of interest (ROI) extracted from one imaging data (sMRI) and two genetics (SNP and gene expression data). The results showed that the algorithm proposed in this paper had better correlation performance. The obtained biomarkers we discovered have diagnostic significance for AD and MCI.

## Method

### MTSCCA

Du et al. put forward MTSCCA [7]. It regarded each image feature as a separate learning task. The objective of MTSCCA is to adjust the typical correlation weights $U \in {R^p}^{ \times M}$ and $V \in {R^p}^{ \times M}$ to maximize the correlation between *Xu*_*m*_ and *Y*_*m*_*v*_*m*_, and its objective function is shown in Formula 1:

1 $$\begin{aligned}&\mathop {\min }\limits_{{u_m},{v_m}} \sum\limits_{m = 1}^M { - u_m^T} {X^T}{Y_m}{v_m} \\& s.t\,\left\| {X{u_m}} \right\|_2^2 = 1,\left\| {{Y_m}{v_m}} \right\|_2^2 = 1,\Omega \left( U \right) \leqslant {b_1},\Omega \left( V \right) \leqslant {b_2}\end{aligned} $$

Where *M* represents the type of modal features of image data, the *m*-th column *v*_*m*_ of weight *V* represents the *m*-th image feature, the *m*-th column *u*_*m*_ of weight *U* represents the weight of SNP corresponding to the *m* -th feature, and *Ω(U)* and *Ω(V)* respectively represent the weight penalty items for SNP and image.

### MTOSCCA

The algorithm proposed in this paper added orthogonal constraints to $\:u$ and $\:v$ on the basis of MTSCCA and applied it to the association analysis of SNP, gene, and sMRI. Given the data set of sMRI, $X \in {R^n}^{ \times p}$, and the genetic data of M modes, ${Y_m} \in {R^n}^{ \times q}\left( {m = 1, \ldots,M} \right)$. Where $\:p$ represents the number of ROI obtained by sMRI, and $\:q$ represents the number of SNP and the number of genes. Its objective function is shown in Formula (2).

2 $$\begin{aligned}&{\mathop {{\text{min}}}\limits_{{\text{U,V}}} \mathop \sum \limits_{{\text{m = 1}}}^{\text{M}} \left( \begin{gathered}\left\| {{\text{X}}{{\text{u}}_{\text{m}}}{\text{ - }}{{\text{Y}}_{\text{m}}}{{\text{v}}_{\text{m}}}} \right\|_{\text{2}}^{\text{2}} + {{{\lambda }}_{{\text{v3}}}}\left\| {{{\text{v}}_{\text{m}}}{{\text{v}}_{\text{m}}}^{\text{T}} - {\text{I}}} \right\|_2^2 \hfill \\+ {{{\lambda }}_{{\text{u2}}}}\left\| {{{\text{u}}_{\text{m}}}{{\text{u}}_{\text{m}}}^{\text{T}} - {\text{I}}} \right\|_2^2 \hfill \\ \end{gathered} \right)} \\& {{\text{ + }}{{{\lambda }}_{{\text{v1}}}}{{\left\| {\text{V}} \right\|}_{{\text{2,1}}}}{\text{ + }}{{{\lambda }}_{{\text{v2}}}}{{\left\| {\text{V}} \right\|}_{{\text{1,1}}}} + {{{\lambda }}_{{\text{u1}}}}{{\left\| {\text{U}} \right\|}_{{\text{2,1}}}}} \\& {{\text{s}}{\text{.t}}{\text{.}}\left\| {{\text{X}}{{\text{u}}_{\text{m}}}} \right\|_{\text{2}}^{\text{2}}{\text{ = 1,}}\left\| {{{\text{Y}}_{\text{m}}}{{\text{v}}_{\text{m}}}} \right\|_{\text{2}}^{\text{2}}{\text{ = 1,}}\forall {\text{m}}} \end{aligned}$$

Among them, *λ*_*v1*_, *λ*_*v2*_, *λ*_*v3,*_*λ*_*u1*_, *λ*_*u2*_ are superparameters that control the sparsity of *V* and *U* and the degree of orthogonal constraint. The expressions of ${\left\| {\text{V}} \right\|_{{\text{2,1}}}}$ and ${\left\| {\text{V}} \right\|_{{\text{1,1}}}}$ are shown in Formula (3) and Formula (4), respectively.

3 $$\:\parallel\:V{\parallel\:}_{\text{1,1}}={\sum\:}_{j=1}^{q}\:{\sum\:}_{m=1}^{M}\:\left|{v}_{jm}\right|$$

4 $${\left\| V \right\|_{2,1}} = \sum\nolimits_{i = 1}^q {{{\left\| {{V_{i,:}}} \right\|}_2}} = \sum\nolimits_{i = 1}^q {\sqrt {\sum\nolimits_{m = 1}^M {V_{i,j}^2} } } $$

To illustrate the derivation details of the CCA class-based algorithm, we present the specific derivation procedure of the CCA algorithm in the Supplementary material. In the penalty term, Eq. 2 can be solved by using Lagrange multiplier method to find the partial derivatives of SNP and the weights *u*_*m*_ and *v*_*m*_ of each brain image modality. First, we consider *U* as a constant term, then the objective function can be rewritten as Eq. (5).

5 $$\begin{gathered}\mathop {\min }\limits_{U,V} \sum\limits_{m = 1}^M {\left( {\left\| {X{u_m} - {Y_m}{v_m}} \right\|_2^2 + {\lambda _{v3}}\left\| {{v_m}{v_m}^T - I} \right\|_2^2} \right)} \hfill \\+ {\lambda _{v1}}{\left\| V \right\|_{2,1}} + {\lambda _{V2}}{\left\| V \right\|_{1,1}} + {\gamma _V}\sum\nolimits_{m = 1}^M {\left\| {{Y_m}{v_m}} \right\|} _2^2 \hfill \\ \end{gathered} $$

For the related task *m*, Eq. 3 differentiates *v*_*m*_ and makes it zero, and Eq. 6 can be obtained.

6 $$\begin{gathered}\:{\text{v}}_{\text{m}}^{\text{T}}{{\text{Y}}_{\text{m}}}{{\text{v}}_{\text{m}}}{\text{ - X}}{{\text{u}}_{\text{m}}} + 2{\lambda _{{\text{v}}3}}({{\text{v}}_{\text{m}}}{{\text{v}}_{\text{m}}}^{\text{T}} - {\text{I}}){\text{u}} \hfill \\{\text{ + }}{\lambda _{{\text{v1}}}}{{\text{D}}_{{\text{v1}}}}{{\text{v}}_{\text{m}}}{\text{ + }}{\lambda _{{\text{v2}}}}{{\text{D}}_{{\text{v2}}}}{{\text{v}}_{\text{m}}}{\text{ + }}\left( {{\gamma _{\text{v}}}{\text{ + 1}}} \right){\text{Y}}_{\text{m}}^{\text{T}}{{\text{Y}}_{\text{m}}}{{\text{v}}_{\text{m}}}{\text{ = 0}} \hfill \\ \end{gathered} $$

The iterative solution formula of *v*_*m*_ can be written as Eq. (7).

7 $$\begin{gathered}\:{{\text{v}}_{\text{m}}}{\text{ = (Y}}_{\text{m}}^{\text{T}}{{\text{Y}}_{\text{m}}}{\text{ + }}{\lambda _{{\text{v1}}}}{{\text{D}}_{{\text{v1}}}}{\text{ + }}{\lambda _{{\text{v2}}}}{{\text{D}}_{{\text{v2}}}}{\text{ + (}}{\gamma _{\text{v}}}{\text{ + 1)Y}}_{\text{m}}^{\text{T}}{{\text{Y}}_{\text{m}}} \hfill \\{\text{ + }}2\lambda {\:_{{\text{v}}3}}({{\text{v}}_{\text{m}}}{{\text{v}}_{\text{m}}}^{\text{T}} - {\text{I}}){{\text{u}}_{\text{m}}}{{\text{)}}^{{\text{ - 1}}}}{\text{(Y}}_{\text{m}}^{\text{T}}{\text{X}}{{\text{u}}_{\text{m}}}{\text{)}} \hfill \\ \end{gathered} $$

Similarly, for *U*, *V* is regarded as a constant term, then the solution formula of *U* can be obtained, as shown in Eq. (8).

8 $$\begin{gathered}\:{\text{U = }}\sum {\:_{{\text{m = 1}}}^{\text{M}}} {\text{(}}{\lambda _{{\text{u1}}}}{{\text{D}}_{{\text{u1}}}}{\text{ + }}{\lambda _{{\text{u2}}}}({{\text{u}}_{\text{m}}}{{\text{u}}_{\text{m}}}^{\text{T}} - {\text{I}}) \hfill \\{\text{ + (}}{\gamma _{\text{u}}}{\text{ + 1)}}{{\text{X}}^{\text{T}}}{\text{X}}{{\text{)}}^{{\text{ - 1}}}}{{\text{X}}^{\text{T}}}{{\text{Y}}_{\text{m}}} \hfill \\ \end{gathered} $$

### Algorithm performance metrics

The canonical correlation coefficient (CCC) was used to measure the performance of the algorithm. Specifically, we refered to the Pearson correlation coefficient in which the expression matrix of sMRI was multiplied by its weight vector U and the expression matrix of SNP was multiplied by its weight vector v1 as CCC1. In addition, the Pearson correlation coefficient when the expression matrix of sMRI was multiplied by its weight vector U and the gene expression matrix was multiplied by its weight vector v2 was defined to as CCC2, and its formulas were shown in Formula (9) and Formula (10).

9 $$\:\text{CCC1=}\text{corr}\text{(X}{\text{u}}_{\text{1}}\text{,}{\text{Y}}_{\text{1}}{\text{v}}_{\text{1}}\text{)}$$

10 $$\:\text{CCC1=}\text{corr}\text{(X}{\text{u}}_{\text{2}}\text{,}{\text{Y}}_{\text{2}}{\text{v}}_{\text{2}}\text{)}$$

Among them, $\:{\text{corr(A}} \cdot {\text{B)}}$ standed for Pearson correlation coefficient between A and B.

### Data preprocessing methods

The genetic and imaging data used in this study were obtained from the Alzheimer’s Disease Neuroimaging Initiative (ADNI) database (https://adni.loni.usc.edu/). The ADNI database provides a substantial amount of data for the research of Alzheimer’s disease (AD) and mild cognitive impairment (MCI), which are derived from multiple phases of data collection, including ADNI1, ADNI-GO/ADNI2, and ADNI3. Each phase has its specific objectives, and the research methods and technologies have also evolved over time. Considering the batch effects of different modalities of data across phases, this study only included samples from the ADNI1 phase. We included 138 non-White participants who had all three types of imaging-genetic data: structural magnetic resonance imaging (sMRI), gene expression profiles, and single-nucleotide polymorphism (SNP) data. The participants comprised 102 patients with MCI and 36 patients with AD. When multiple time points of imaging data were available for a sample, the most recent measurement was prioritized as a reference to ensure that the analyzed data could maximally reflect the current status of the participants and avoid biases caused by large time intervals. All included samples were required to have completed the Mini-Mental State Examination (MMSE) to assess their cognitive function status. Additionally, the imaging data were required to be clear and free of artifacts, accurately reflecting the structural and functional information of the brain. Samples with missing data or quality issues were excluded.

The specific information of all experimental samples in this chapter is shown in Table 1. We divided all the samples into 8:2 and randomly divided the training set and the test set. Finally, 110 training set samples (including 30 AD and 80 MCI samples) and 28 test set samples (6 AD and 22 MCI samples) were obtained.

**Table 1:** Specific statistical information of experimental samples

Groups	AD	MCI
Number	36	132
Gender(M/F)	20/16	70/62
Age(mean ± std)	77.55 ± 9.86	72.95 ± 8.07

For the preprocessing of sMRI, this paper first standardized the brains of all subjects to the standard space of the Montreal Institute of Neurology by SPM toolkit [8], and the voxel signal intensity will be corrected with the transformation. Then the gray matter was segmented using the human brain tissue probability map. Then it was registered to the AAL brain map, and the ROI expression matrix of 90 brain regions with the cerebellar area removed was extracted and arranged as sMRI.

In order to get the characteristic data of SNP, the PLINK tool was applied to preprocess the genotype data [9]. According to the standard process, we removed SNPs that do not meet the standards of sex detection and Hardy-Weinberg equilibrium (the P value was less than 10 − 6), and the frequency of secondary alleles was less than 0.05. In addition, SNP data were genotyped by Michigan Impressions Server with HRC r1.1 2016 as the reference sequence set, and the filled data were filtered with R squared greater than 0.3, which ensured the filling quality. After that, ANNOVAR was used to annotate the filled SNPs, and 2380 SNPs were extracted in 5 K base pairs at the boundaries of 13 AD risk genes (CR1, MEF2C, CELF1, MS4A6A, SORL1, FERMT2, SLC24A4, RIN3, ADAM10, ABCA7, APOE, CD33, and CASS4). For the gene expression matrix, we obtained 413 differentially expressed genes of AD and MCI with *p* < 0.05 by the Limma algorithm for subsequent analysis.

### Random forest algorithm screening diagnosis-related SNP

The MTOSCCA algorithm requires an equal number of genes and SNPs as input. To meet this requirement while preserving the most informative genetic features, we employed the Random Forest (RF) algorithm for feature selection. Prior to its application, we conducted a comparative evaluation of several commonly used machine learning methods, including Support Vector Machine (SVM) and Logistic Regression (LR), in our preliminary experiments. We found that RF outperformed these models in both feature importance stability and predictive performance on our dataset. Specifically, RF provided consistent feature importance scores across multiple runs, whereas SVM and LR exhibited greater variability. In cross-validation and independent testing, RF achieved higher accuracy (0.84) and AUC (0.62) compared to SVM (accuracy: 0.78, AUC: 0.59) and LR (accuracy: 0.75, AUC: 0.59). The ensemble nature of RF enables it to better capture complex nonlinear relationships and offers robustness against noise and outliers. Therefore, we applied RF using the “GridSearchCV” function from the scikit-learn package in Python to select a subset of SNPs that matches the number of genes. Feature selection was performed solely on the training set to prevent information leakage from the test set. The selected SNPs, together with the corresponding genes, were subsequently used as inputs for the MTOSCCA algorithm to identify significant gene-SNP associations.

## Results

### Parameter selection and results of the algorithm

**Fig. 1:**
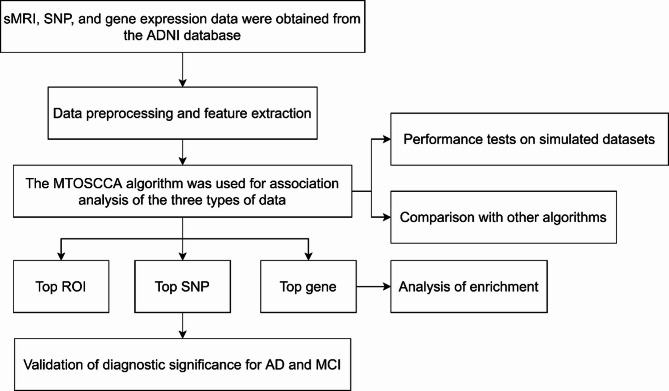
The overall flow chart of the paper

**Fig. 2:**
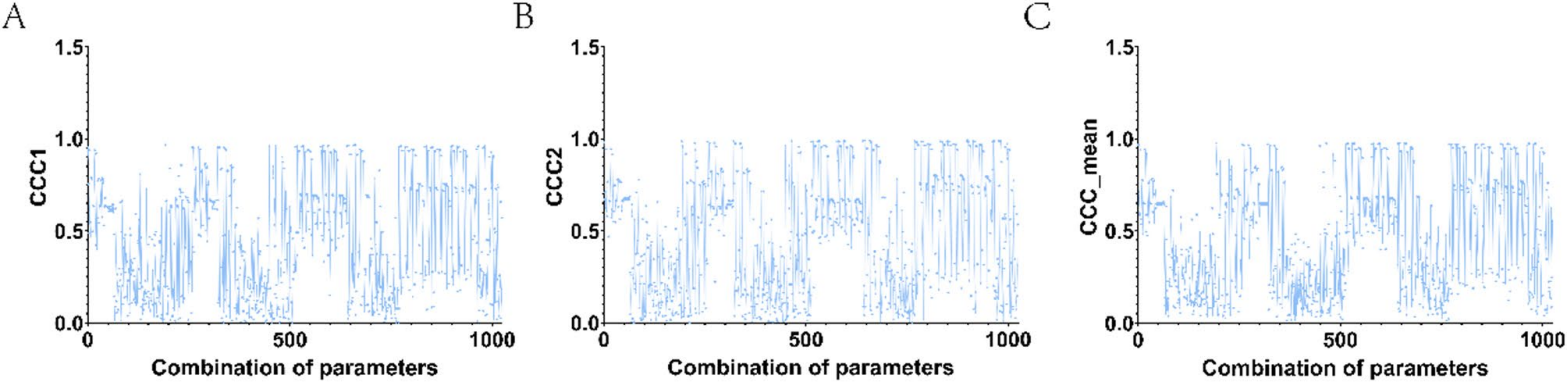
Selection of optimal parameters. **A** and **B** were the change line charts of CCC1 and CCC2 under different parameter combinations, respectively. **C** was the change line chart of the mean value of CCC1 and CCC2 under different parameter combinations

**Fig. 3:**
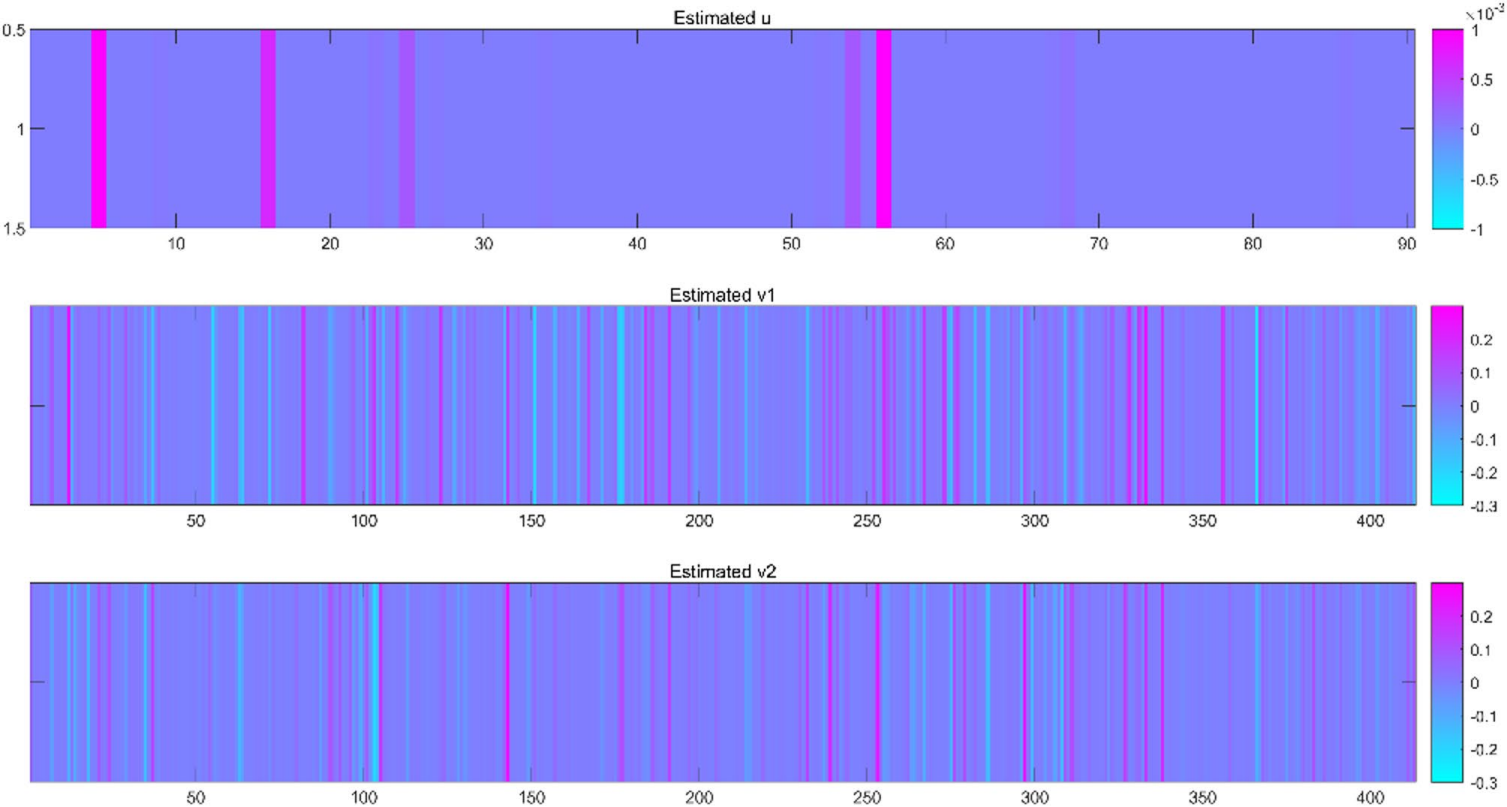
Weighted heat map of $\:\text{u}$, $\:{\text{v}}_{\text{1}}$ and $\:{\text{v}}_{\text{2}}$

The overall flow chart of the paper was given in Fig. 1. In this paper, firstly, the weight information of all SNPs was obtained by the RF algorithm and sorted from large to small, and the expression of the first 413 SNPs was reserved for subsequent analysis. Parameters need to be selected for the MTOSCCA algorithm include *λ*_*v1*,_*λ*_*v2*,_*λ*_*v3*,_*λ*_*u1*,_ and *λ*_*u2*_. We chose from the range of parameter set [0.001 0.01 0.1 1] by using the strategy of grid search. We show the corresponding values of taking the absolute values of CCC1 and CCC2 and their mean values under 1024 parameter combinations in Tables S1-S3, respectively. We displayed the absolute values of CCC1 and CCC2 and the changes in their mean values under different parameter combinations in Fig. 2. As shown in Fig. 2C, the largest average CCC was obtained in the 658th iteration. Therefore, we take the 658th parameter combination (*λ*_*v1*__*v2*_ = 0.001, *λ*_*v3*_ = 0.01, *λ*_*u1*_ = 0.1和*λ*_*u2*_ = 0.1) as the optimal parameter combination. Finally, we gave the weight heat map of $\:\text{u}$,$\:{\text{v}}_{\text{1}}$ and $\:{\text{v}}_{\text{2}}$ under this combination (Fig. 3). The discussion section will explore the relationship among the selected Top ROI, Top SNP, Top gene, and AD in detail.

### 3.2 Simulation data set algorithm performance test

**Fig. 4:**
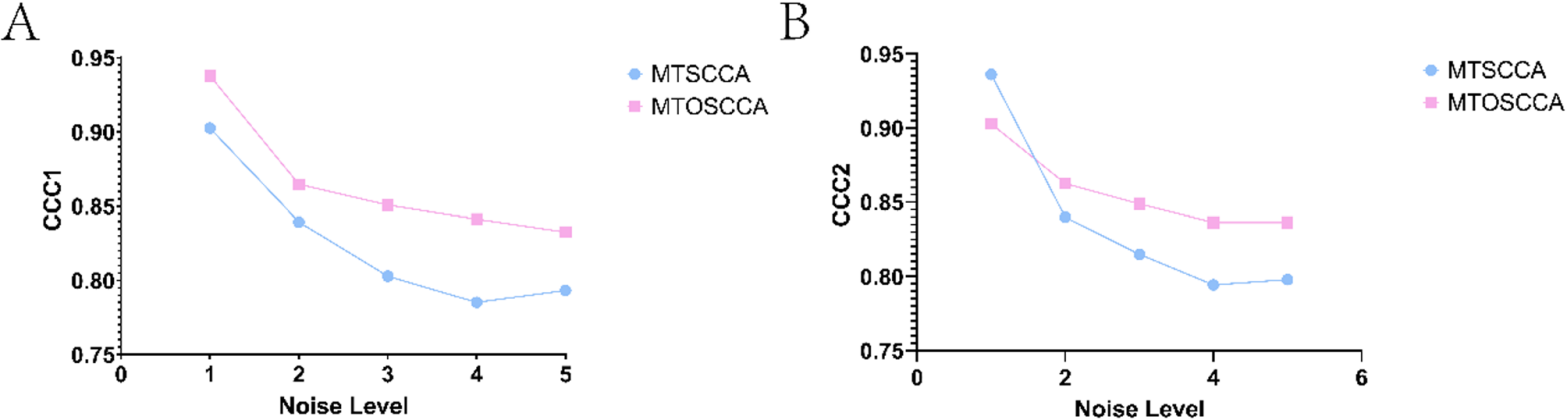
Performance comparison results of two algorithms on simulated data sets. **A** and **B** were the comparisons of CCC1 and CCC2 of the two algorithms under different noises, respectively

In this section, we tested the performance of the MTSCCA algorithm and MTOSCCA algorithm on simulated data sets. We used the matrices $X \in {R^n}^{ \times p}$, ${Y_1} \in {R^n}^{ \times q}$ and ${Y_2} \in {R^n}^{ \times q}$ to represent the expression matrices of ROI, SNP, and gene, respectively. X can be calculated by formula *X = uc + e*, Y1 can be obtained by formula *Y*_*1*_ *= v*_*1*_*c + e*, and Y2 can be obtained by formula *Y*_*2*_ *= v*_*2*_*c + e*. Among them, U, v1, and v2 are the known typical correlation coefficient vectors. Vector E conforms to Gaussian distribution and is the noise variance, which can be used to control the noise in data. In order to test the performance of the model under different noise levels, the tolerance is increased from 1 to 5, and there are five different noise levels. Set *n* = 100, *p* = 90 and q = 650. In Fig. 4, we showed the size changes of CCC1 and CCC2 of the two algorithms under different noises. As can be seen from the figure, the proposed algorithm has better anti-noise performance.

### Diagnostic significance of top markers

**Fig. 5:**
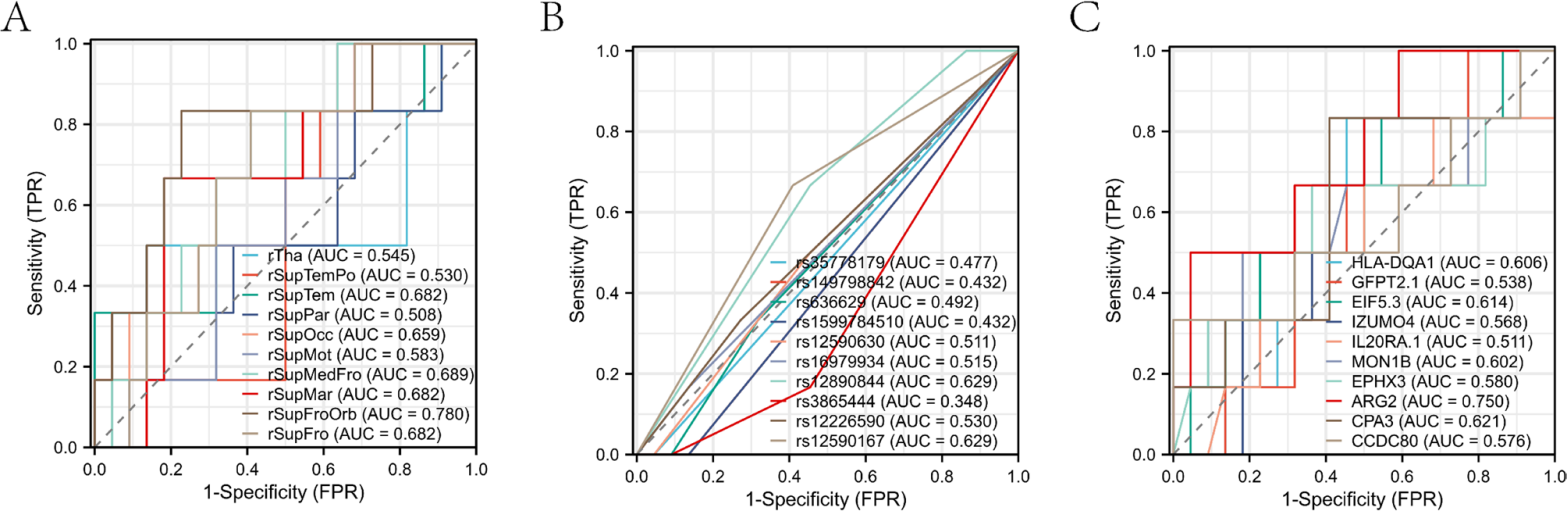
The ROC curve of the Top biomarker. **A**-**C** were the ROC curves of the Top 10 ROI, SNP, and gene, respectively

To further assess the robustness and generalizability of our findings using the MTOSCCA algorithm, we employed a five-fold cross-validation method. This method involved dividing the dataset into five subsets, with four subsets used for training and the remaining one for validation. This process was repeated five times, each time using a different subset for validation. We gave the name and weight information of the Top 10 ROI, SNP, and gene in Table 2, and we will discuss in detail the critical role of these markers in the occurrence and development of AD in the discussion section. In addition, we showed the diagnostic significance of these markers for AD and MCI by ROC curve in Fig. 5 and gave the specific information of ROC curve on the test set in Tables 3, 4 and 5, respectively. As can be seen from the figure, the AUC of all the Top ROI, Top gene, and half of the Top SNP were all greater than 0.5 and all within a reasonable confidence interval. Finally, we also validated the joint predictive performance of the top (combined) features from each modality. The combined predictive performance of the three modalities (Table 6) achieved the best result (AUC = 0.955). Finally, this paper also compared the performance of the MTOSCCA and MTSCCA algorithms. Specifically, the CCC of the MTOSCCA algorithm was 0.347 (CI: 0.234–0.362), while that of the MTSCCA algorithm was 0.316 (CI: 0.205–0.437). The AUC values for the top features selected by MTSCCA are presented in Tables S4–S5 of the supplementary material.

**Table 2:** Top marker names and their weights

ROI	Weights	SNP	Weights	Gene	Weights
rSupTemPo	3.92E-08	rs149798842	0.333478	GFPT2.1	0.277304
rSupTem	1.98E-05	rs636629	0.262529	EIF5.3	0.250054
rSupPar	1.32E-08	rs1599784510	0.248188	IZUMO4	0.235172
rSupOcc	9.72E-09	rs12590630	0.209298	IL20RA.1	0.214808
rSupMot	8.06E-06	rs769485	0.196871	MON1B	0.205919
rSupMedFro	7.41E-06	rs12890844	0.188306	EPHX3	0.197324
rSupMar	3.06E-06	rs3865444	0.18622	ARG2	0.177393
rSupFroOrb	1.41E-06	rs429358	0.184627	CPA3	0.170478
rSupFro	2.90E-06	rs12590167	0.18238	CCDC80	0.154335
rTha	7.02E-09	rs35778179	0.097301	HLA-DQA1	0.054587

Note: rTha = Right Thalamus, rSupTemPo = Right Superior Temporal Pole, rSupTem = Right Superior Temporal, rSupPar = Right Superior Parietal, rSupOcc = Right Superior Occipital, rSupMot = Right Superior Motor, rSupMedFro = Right Superior Medial Frontal, rSupMar = Right Supramarginal, rSupFroOrb = Right Superior Frontal Orbital, rSupFro = Right Superior Frontal

**Table 3:** Specific information of ROC curve of top rois on the test set

Predictive variable	Area Under Curve (AUC)	Confidence Interval (CI)
rTha	0.545	0.187–0.903
rSupTemPo	0.53	0.276–0.785
rSupTem	0.682	0.405–0.959
rSupPar	0.508	0.228–0.787
rSupOcc	0.659	0.414–0.904
rSupMot	0.583	0.351–0.816
rSupMedFro	0.689	0.466–0.913
rSupMar	0.682	0.447–0.917
rSupFroOrb	0.78	0.548–1.000
rSupFro	0.682	0.460–0.904

**Table 4:** Specific information of ROC curve of top SNPs on the test set

Predictive variable	Area Under Curve (AUC)	Confidence Interval (CI)
rs35778179	0.477	0.433–0.522
rs149798842	0.432	0.358–0.505
rs636629	0.492	0.274–0.710
rs1599784510	0.432	0.358–0.505
rs12590630	0.511	0.273–0.750
rs16979934	0.515	0.336–0.694
rs12890844	0.629	0.415–0.842
rs3865444	0.348	0.163–0.534
rs12226590	0.53	0.271–0.790
rs12590167	0.629	0.397–0.861

**Table 5:** Specific information of ROC curve of top genes on the test set

Predictive variable	Area Under Curve (AUC)	Confidence Interval (CI)
HLA-DQA1	0.606	0.373–0.839
GFPT2.1	0.538	0.282–0.793
EIF5.3	0.614	0.340–0.888
IZUMO4	0.568	0.288–0.848
IL20RA.1	0.511	0.226–0.797
MON1B	0.602	0.288–0.917
EPHX3	0.58	0.267–0.892
ARG2	0.75	0.519–0.981
CPA3	0.621	0.342–0.901
CCDC80	0.576	0.256–0.895

**Table 6:** Specific information of ROC curve of combined feature on the test set

Predictive variable	Area Under Curve (AUC)	Confidence Interval (CI)
Top 10 ROIs	0.773	0.512–0.970
Top 10 genes	0.765	0.479–0.974
Top 10 SNPs	0.837	0.640–0.973
Top 10 ROIs + Top 10 genes	0.803	0.586–0.963
Top 10 ROIs + Top 10 SNPs	0.902	0.735–1
Top 10 genes + Top 10 SNPs	0.909	0.774–1
Top 10 ROIs + Top 10 genes + Top 10 SNPs	0.955	0.867–1

## Discussion

To integrate the sMRI, SNP, and gene expression data of AD and MCI, this study proposes an MTOSCCA algorithm. By introducing orthogonality constraints into the weight vectors, this algorithm effectively addresses the issue of feature redundancy. Specifically, in the context of multimodal data integration, redundant features often refer to those that are highly correlated and capture the same biological processes without providing additional information. For example, multiple highly correlated SNPs or genes may reflect the same genetic variation or biological mechanism. By imposing orthogonality constraints on the weight vectors $\:\mathbf{U}$ and $\:\mathbf{V}$, the MTOSCCA algorithm ensures that the selected features are linearly independent in a mathematical sense. This means that each feature contributes unique information to the correlation analysis, thereby reducing redundancy. Specifically, the orthogonality constraints $\:{\mathbf{u}}_{m}{\mathbf{u}}_{m}^{T}-\mathbf{I}$ and $\:{\mathbf{v}}_{m}{\mathbf{v}}_{m}^{T}-\mathbf{I}$ in the objective function guarantee the orthogonality of the weight vectors, thus preventing the selection of highly correlated features. This orthogonality constraint not only enhances the feature selection capability of the algorithm but also improves the stability and generalizability of the model.

**Fig. 6:**
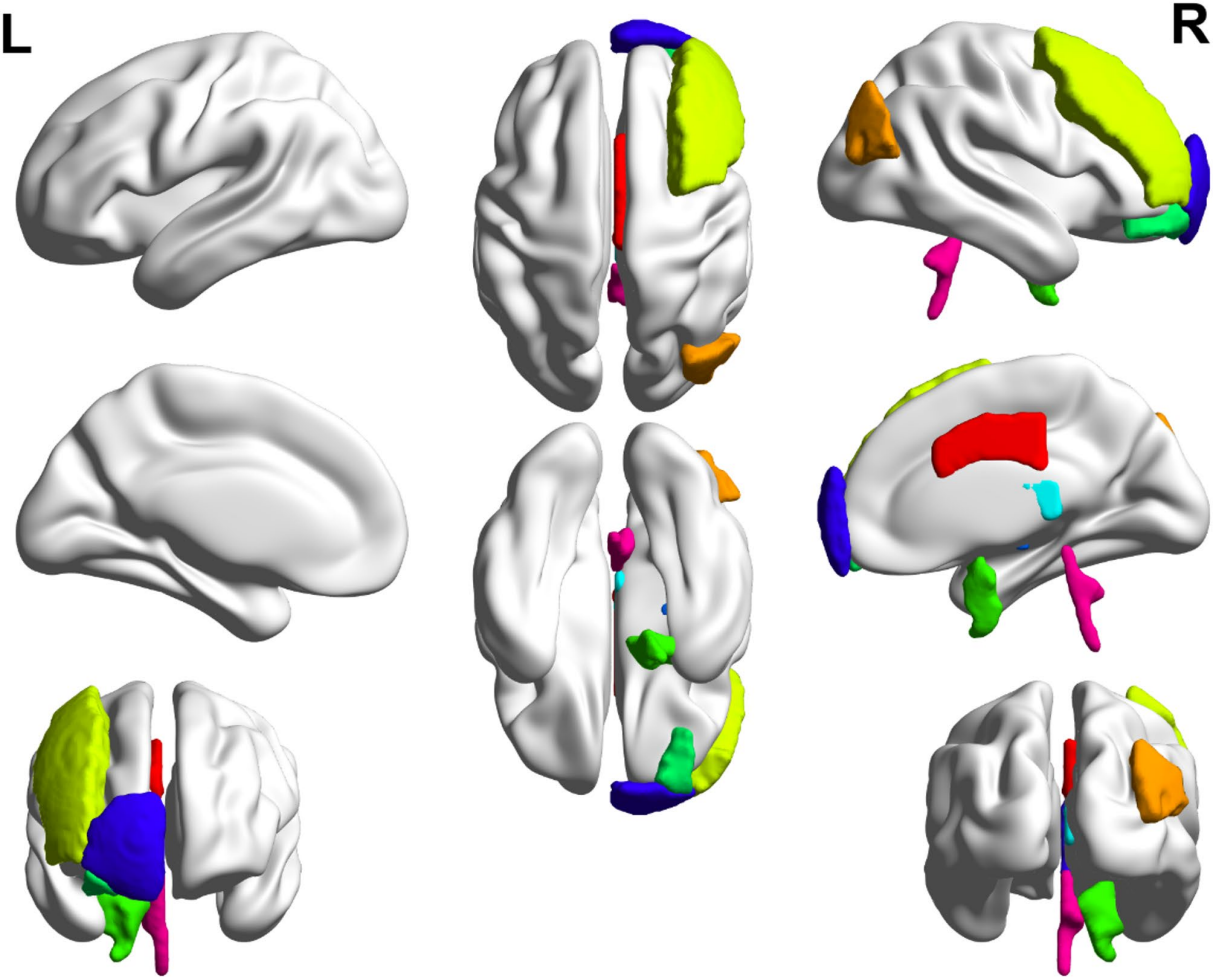
Visualization of the Top brain region

The results on real data sets showed that most of the Top markers selected by the algorithm have diagnostic significance for AD and MCI. We offered the visualization of the Top brain region in Fig. 6. Feng F et al. found that the structural connection between the left hippocampus and thalamus will affect their functional connectivity (FC) in patients with AD and aMCI [10]. Compared with non-depressed AD patients (nD-AD), the lymph node centrality of depressed AD patients (D-AD) decreased in the right superior temporal gyrus and increased in the right superior parietal gyrus [11]. Compared with HC, FC in AD patients’ medial frontal gyrus and superior frontal gyrus decreased [12].

From a biological perspective, the introduction of orthogonality constraints is of great significance. Some genes in the Top 10 gene have also been confirmed to be closely related to AD. We determined that HLA-DQA1, IL20RA.1, EPHX3 and CCDC80 are directly or indirectly related to AD. AD affects the expression of HLA-DQA1 in human microglia [13]. IL20RA.1 is a member of the IL20RA family. The related pathways of IL-20 RA include Akt signaling and cytokine signaling in the immune system. TREM2 improves neuroinflammatory reactions and cognitive impairment in AD mice through PI3K/AKT/FoxO3a signaling pathway [14]. EPHX3 gene makes epoxide hydrolase active and participates in epoxide metabolism. Recent studies have shown that epoxide hydrolase inhibitors reduce neuroinflammation in mouse models of Alzheimer’s disease [15]. CCDC80 gene can promote cell adhesion, and investigations by Ilic K et al. showed that cell adhesion protein neurotic AD is an early potential biomarker [16].

**Fig. 7:**
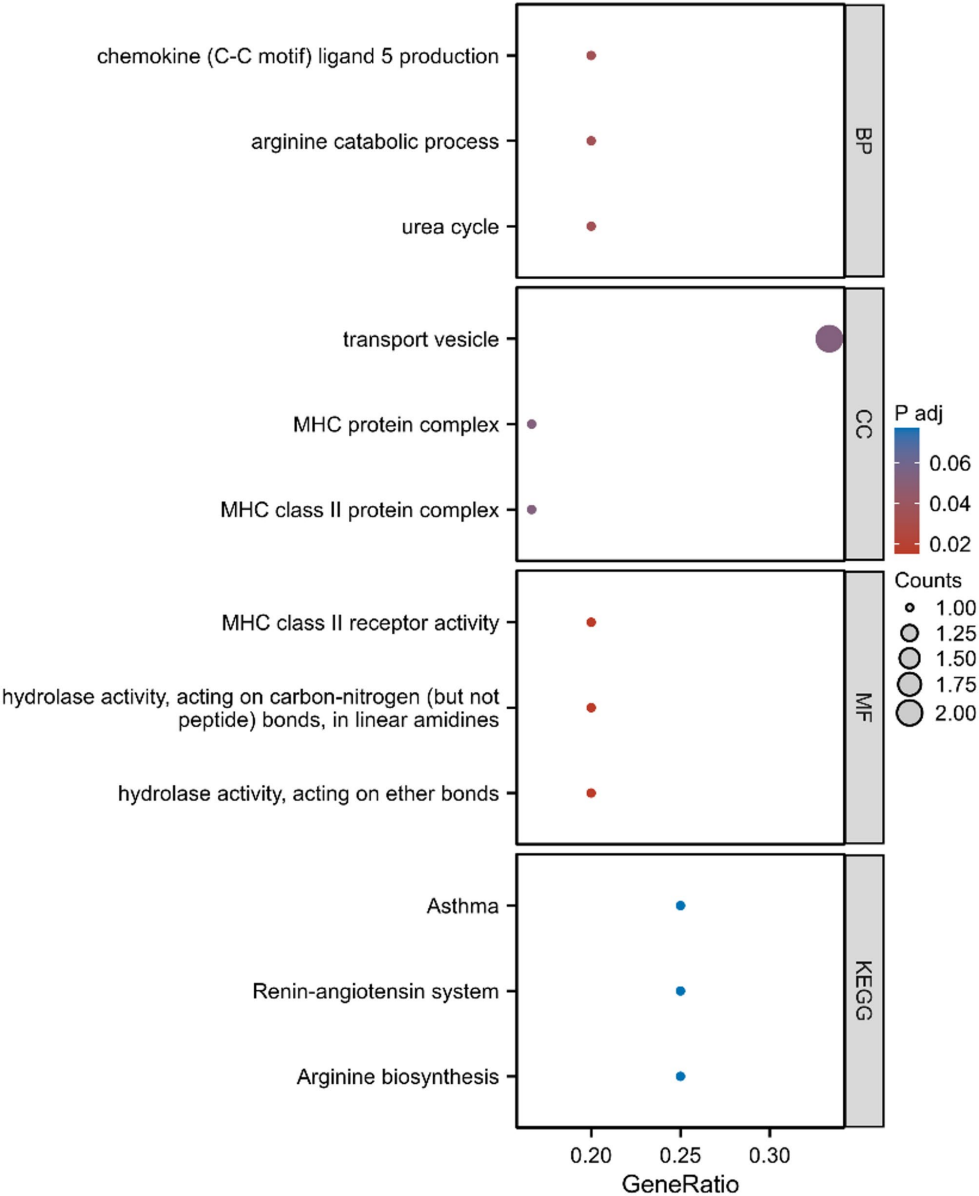
Enrichment and analysis results of GO and KEGG of the Top gene

In terms of diagnostic value, the biomarkers identified through the orthogonality constraints demonstrated significant diagnostic power in differentiating AD, MCI, and healthy controls. The results of the ROC analysis showed that the AUC of the combined features reached 0.955, indicating a high level of diagnostic accuracy for these biomarkers. This not only validates the biological relevance of the selected biomarkers but also highlights their potential clinical application value. By reducing redundancy, the orthogonality constraints help to construct a more robust and generalizable model, which is crucial for clinical applications, as the model needs to perform well on unseen data. In addition, we took GO and enrichment analysis on the Top 10 gene obtained by the algorithm (Fig. 7). Most of these pathways have been proven to be closely related to the occurrence and development of AD. During the change of AD, the activity and protein expression of arginase will also change, and arginine metabolism will also change significantly in the AD brain [17, 18]. Aβ is an important factor causing AD, and astrocytes can absorb and remove Aβ plaque [19, 20]. The recent research results of Ju YH et al. show that the urea cycle of astrocytes can detoxify Aβ and damage the memory of AD [21]. The major histocompatibility complex (MHC) polymorphism has always been the focus of plenty of AD association studies [22]. Microglia usually present T-cell antigens through class II MHC, which plays a vital role in AD [23]. Targeting the renin-angiotensin system in the brain may have positive significance for AD patients [24]. The role of the Top 10 SNP in the occurrence and development of AD needs further study.

This study has made some progress in exploring the imaging-genetic features of AD and MCI, but there are also some limitations. First, since the imaging-genetic features of the control group are significantly different from those of MCI and AD, and distinguishing between MCI and AD is of great significance in clinical practice, this study did not include a healthy control group. This limits the direct comparison of differences between normal aging and pathological processes. Second, the imbalance in the ratio of MCI to AD participants may affect the algorithm’s performance, causing it to bias towards the majority class (MCI) during training, thereby affecting its ability to recognize the minority class (AD). Although we assessed and mitigated this impact through random division of training and test sets, multiple repetitions of experiments, and optimization of algorithm parameters, the imbalance in sample ratios remains an issue that needs to be addressed. Moreover, the APOE gene or its variants did not appear among the top SNPs or genes, despite being the most widely validated genetic risk factor for AD. This may be related to the limitations of the algorithm, the ethnic background and disease stage of the samples, omissions in the feature selection process, and the fact that the influence of the APOE gene on AD may be more prominently manifested at the functional connectivity or metabolic level. In future studies, we will prioritize the inclusion of a healthy control group to more comprehensively evaluate the biomarker patterns of HC (healthy controls), MCI, and AD. We will also address the sample ratio imbalance by employing data resampling techniques such as SMOTE, adjusting algorithm weights, and increasing the sample size. Additionally, we will further optimize the algorithm and data processing procedures, expand the sample size, incorporate functional imaging data, and attempt to integrate clinical information as prior knowledge into the algorithm. This will allow for a more comprehensive assessment of the relationship between the APOE gene and AD-related features, providing stronger support for the early diagnosis and treatment of AD.

## Conclusion

In this paper, an MTOSCCA algorithm was proposed and applied to integrate the image genetic data of AD. This algorithm has detected many biomarkers with biological and diagnostic significance. In future research, we will try to add clinical information as a priori knowledge to the algorithm to obtain more clinically interpretable results.

## Electronic supplementary material

Below is the link to the electronic supplementary material.

Supplementary Material 1

Supplementary Material 2

## Data Availability

The data in this paper came from ADNI database (https://adni.loni.usc.edu/).

## Code availability

In this paper, the proposed MTOSCCA algorithm code can be obtained at https://github.com/yinwei1990/MTOSCCA.
