# Supplementary material for: Exploration of biomarkers of Alzheimer’s disease based on orthogonal multi-task canonical correlation analysis
Source: BMC Med Imaging. 2025 Jul 17;25:287. doi: 10.1186/s12880-025-01782-2 (PMC12272997; doi:10.1186/s12880-025-01782-2)
Supplement: Supplementary file 1 — Supplementary Material 1 [file 12880_2025_1782_MOESM1_ESM.docx]

**The concrete derivation process of CCA algorithm**

Given two expression matrices *X∈R^n×p^* and *Y∈R^n×q^*, where n represents the number of samples, p and q represent the characteristic numbers of the two expression matrices, respectively. CCA algorithm maps *Xu∈R^n×1^* and *Yv∈R^n×1^* to n-dimensional space, that is, *Xu∈R^n×1^* and *Yv∈R^n×1^* through transformation matrices *X∈R^n×p^* and *Y∈R^n×q^*. This linear transformation can make the included angle *θ*（*0≦θ≦π*）between *Xu* and *Yv* minimum and the cosine maximum, thus the objective function of CCA can be obtained:

$\begin{matrix} cos\theta=\mathop{max}\limits_{u,v} \left\langle Xu,Yv\right\rangle=\mathop{max}\limits_{u,v} u^{T}X^{T}Yv \\ s.t.\mathbf{ }\left\|Xu\right\|_{2}^{2}=u^{T}X^{T}Xu=1, \left\|Yv\right\|_{2}^{2}=v^{T}Y^{T}Yv=1 \end{matrix}$ (1)

Lagrange multiplier method can solve the optimization problem of CCA objective function in formula (1), and it can transform formula (1) into formula (2).

$L=u^{T}X^{T}Yv-\frac{\rho_{1}}{2}\left(u^{T}X^{T}Xu-1\right)-\frac{\rho_{2}}{2}\left(v^{T}Y^{T}Yv-1\right)$ (2)

Then, we take the partial derivatives of u and v respectively and make the result 0. The formulas (3)-(4) can be obtained.

$\frac{\partial L}{u}=X^{T}Yv-\rho_{1}X^{T}Xu=0$ (3)

$\frac{\partial L}{v}=Y^{T}Xu-\rho_{2}Y^{T}Yv=0$ (4)

Further, let the formula (3) left multiplied by *u^T^* and the formula (4) left multiplied by *v^T^*, the formulas (5)-(6) can be further obtained.

$u^{T}X^{T}Yv-\rho_{1}u^{T}X^{T}Xu=0$ (5)

$v^{T}Y^{T}Xu-\rho_{2}v^{T}Y^{T}Yv=0$ (6)

Because *u^T^X^T^Xu=1* and *v^T^Y^T^Yv=1*, we can get ρ1=ρ2=ρ. Then, formulas (7)-(8) can be obtained.

$u=\frac{(X^{T}X)^{-1}X^{T}Yv}{\rho}$ (7)

$\frac{1}{\rho}Y^{T}X(X^{T}X)^{-1}X^{T}Yv-\rho Y^{T}Yv=0$ (8)

It can be clearly seen that the optimization of CCA's objective function becomes a generalized eigenvalue problem, which can be easily solved:

$Y^{T}X(X^{T}X)^{-1}X^{T}Yv=\rho^{2}Y^{T}Yv$ (9)

If the matrix *X^T^X* is invertible, it is further transformed into the problem of finding standard eigenvalues:

$(Y^{T}Y)^{-1}Y^{T}X(X^{T}X)^{-1}X^{T}Yv=\rho^{2}v$ (10)
